# Assessment of the tribological behaviour of self-lubricating metal matrix composites developed for circular saw guide pad applications

**DOI:** 10.1186/s40712-026-00481-2

**Published:** 2026-05-26

**Authors:** A. Torkghashghaei, S. Gélinas, C. Blais

**Affiliations:** Department of Mining, Metallurgical and Materials Engineering, Université Laval, 1065 de La Médecine, Quebec City, QC G1V 0A6 Canada

**Keywords:** Cu–Fe-based composite, Powder metallurgy, Self-lubricating materials, Guide pad, Wear resistance, Tribological performance, Surface morphology

## Abstract

The development and performance of self-lubricating Cu-Fe-based composites produced via powder metallurgy are examined as an advanced alternative to conventional Babbitt alloy for guide pad applications in high-speed circular saw systems. The proposed composite addresses the inherent limitations of Babbitt, particularly its low mechanical strength and poor wear resistance under high contact pressures, aiming to improve durability and operational reliability. A baseline Cu-5 vol.% Ni-coated graphite (NCG)-6 vol.% CaF₂ composite was reinforced with incremental Fe additions (12–52 vol.%) to enhance mechanical strength while preserving favorable tribological properties. The composites were characterized through pin-on-disk and dry sand rubber wheel (DSRW) wear tests, complemented by mechanical evaluation (hardness and tensile testing) and detailed microstructural examination using SEM/EDS. The results demonstrate that increasing Fe content significantly improves hardness, yield strength, and wear resistance, with the composite containing 42 vol.% Fe achieving the optimal performance balance. This composition exhibited nearly a twofold increase in hardness and an approximately threefold increase in yield strength, along with a 94% reduction in sliding wear during pin-on-disk test and an 81% reduction in abrasive wear compared to Babbitt. These improvements are attributed to the enhanced load-bearing capacity of the Fe-reinforced matrix and the formation of a stable oxide-rich tribolayer that effectively limits direct surface interaction and suppresses material removal. In contrast, excessive Fe content (52 vol.%) led to brittle oxide formation and increased wear due to crack propagation and delamination, confirming the existence of a critical reinforcement threshold. Semi-industrial testing further validated the superior performance of the optimized Cu-Fe-based composite. Compared to Babbitt, the optimized composite demonstrated reduced power consumption and negligible material transfer to the saw blade, indicating improved tribological efficiency. Surface morphology analyses of the guide pads revealed that while Babbitt guide pad experienced severe material loss, the Cu-Fe-based composite maintained a smooth and coherent contact surface with minimal damage. Overall, the Cu-42 vol.%Fe-5 vol.%NCG-6 vol.%CaF_2_- composite emerges as a highly effective and durable replacement for Babbitt guide pads, offering superior wear resistance.

## Introduction

In high-speed circular sawing operations, precise blade alignment is essential to ensure dimensional accuracy, extend tool life, and prevent mechanical failure. During cutting, the saw blade is subjected to lateral forces that may disturb its path if not properly controlled (Ding et al. 2023). These disturbances are often caused by irregular wood movements, such as lateral displacement or sudden acceleration of the workpiece within the cut. Such instability increases the likelihood of blade deflection, vibration, or even permanent damage, ultimately compromising the quality of the cut and the safety of the process (Yan et al. 2023).

To counteract these effects, guided circular saw systems are employed to provide enhanced lateral support within the cutting zone (Schajer 1986; Khorasany et al. 2012). Guide pads restrict transverse blade motion and enable the blade to withstand lateral cutting forces, ensuring a stable cutting path and consistent cut uniformity. Nevertheless, dynamic loads generated by residual stresses in the wood or external vibrations can impose substantial forces on the guide pads. When these loads exceed the mechanical or tribological limits of the guiding system, accelerated wear or deformation of the pads may occur, leading to reduced service life and a gradual loss of cutting stability (Orlowski et al. 2022).

**Fig. 1:**
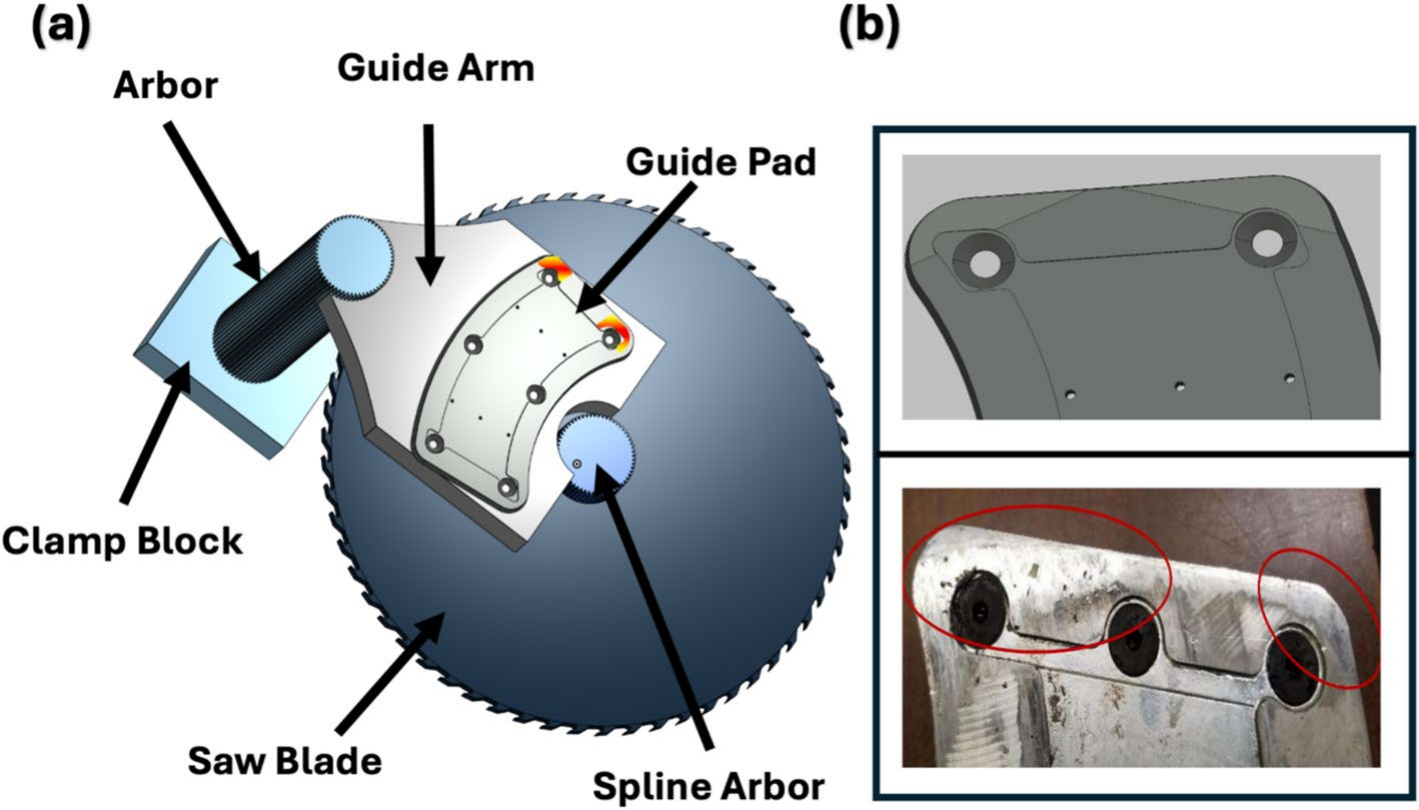
**a** Circular guided saw component, (**b**) Regions with the highest susceptibility for wear and deformation

Wood movement during cutting cannot be completely eliminated unless the wood is held perfectly rigid. Even slight shifts or deviations can negatively impact the guide pad’s performance, causing failure. The guide pad in a circular saw is commonly manufactured from Babbitt alloy through a casting process. Although Babbitt is well known for its excellent tribological performance and is widely used as an anti-friction material in engineering applications (Korshunov et al. 2009), it tends to experience considerable wear and deformation when employed as a guide pad. Babbitt alloys are intrinsically soft and have low mechanical properties, making them highly prone to failure under mechanical loads (Luo et al. 2024). Babbitt alloy possesses inherently low yield strength at room temperature, and its load-bearing capability declines even further as temperature increases. When the temperature increases to around 100 °C, however, its yield strength decreases dramatically (Sadykov et al. 2002). This notable decline with rising temperature highlights Babbitt’s poor thermal stability and its tendency to deform under high-temperature conditions (Sadykov et al. 2002). Figure 1 depicts the circular saw and its guiding system. The regions highlighted in red/yellow correspond closely to the areas most commonly affected by wear or deformation on the Babbitt guide pad in a real sawmill operating condition.

Previous studies have investigated the influence of solid lubricants on the tribological performance of Cu-based composites (Moustafa et al. 2002; Chen et al. 2008; Moazami-Goudarzi and Nemati 2018; Li et al. 2023). In previous work, it was demonstrated that a composite comprising 89 vol.% Cu, 5 vol.% nickel-coated graphite (NCG), and 6 vol.% calcium fluoride (CaF₂) achieved a 68% reduction in the coefficient of friction (COF) and a 67% decrease in wear rate compared with a conventional Babbitt alloy during pin-on-disk testing under a 5 N applied load (Torkghashghaei et al. 2025). The same study further showed that, under three-body abrasion conditions, the wear loss was reduced by 46% relative to Babbitt.

This optimized composite exhibited a highly stable COF and consistently low wear rates, which were attributed to the combined effect of NCG and CaF₂. The presence of NCG facilitated the formation and retention of a lubricious, carbon-rich transfer film at the sliding interface, thereby reducing shear resistance during contact. Simultaneously, CaF₂ contributed to interfacial stabilization by limiting direct metal-to-metal contact and mitigating material removal during sliding. Owing to this favorable and stable tribological response, the Cu-5 vol.% NCG-6 vol.% CaF₂ formulation was identified as an optimized baseline composition and was therefore selected for further reinforcement in the present study.

Cu-based friction materials are well known for their excellent thermal conductivity and high density, which are advantageous in high-speed applications (Zhang et al. 2018). However, Cu-based composites typically exhibit relatively low mechanical strength (Zhai et al. 2024), which limits their wear resistance under severe tribological conditions. Several studies have reported that pure copper and Cu-rich systems suffer from severe plastic deformation and adhesive wear during dry sliding due to their low hardness and high ductility (Zheng et al. 2019).

In comparison, iron-based friction materials offer outstanding mechanical strength and thermal resistance, making them highly suitable for applications involving elevated loads and temperatures. Nonetheless, under such conditions, they are prone to adhesive interactions with the counter surface, which can deteriorate their tribological behavior. In addition, the formation of hard and brittle iron oxides during sliding can introduce abrasive third-body particles, further increasing friction and wear instability (Stott 1998). These factors collectively limit the overall tribological performance of iron-based materials despite their superior mechanical and thermal properties.

To overcome these limitations, Cu-Fe-based composites integrate the beneficial characteristics of both copper and iron, maintaining copper's excellent thermal conductivity capabilities, while iron reinforcement markedly enhances their mechanical strength, wear resistance, and ability to withstand high loads (Zheng et al. 2019). This synergistic effect arises from the combination of a ductile Cu matrix, which ensures efficient heat dissipation and conformability, and hard Fe particles that act as load-bearing constituents, thereby reducing localized plastic deformation during sliding. Recent studies have shown that incorporating Fe into Cu-based composites enables a more favorable balance between mechanical strength, electrical/thermal conductivity, and wear resistance (Sakhaei et al. 2025).

Increasing the iron content in copper-based composites leads to a notable decrease in wear loss (Venkateswaran et al. 2007; Zhou et al. 2019). This improvement is primarily attributed not only to the reinforcing effect of iron, but also to its role in modifying the wear mechanism from severe adhesive wear to more oxidative or mild abrasive wear regimes (Zheng et al. 2019). Fe addition can further promote the formation of a protective tribo-oxide layer during sliding, which reduces direct metal–metal contact and enhances surface stability (Yu et al. 2021). However, it is important to note that the beneficial effects of Fe are highly dependent on its content. While moderate Fe additions contribute to stabilizing the coefficient of friction and minimizing wear loss (Oke et al. 2017), excessive Fe content may lead to increased friction and wear instability due to the formation of brittle oxide debris and enhanced interaction with the counterface (Quinn 1983).

This work explores the effect of incremental Fe additions on a self-lubricating Cu-based composite containing 5 vol. %NCG and 6 vol.% CaF₂. By tailoring the Fe content, the study seeks to boost the composite’s mechanical strength and tribological behavior, ultimately producing a material that outperforms traditional Babbitt alloy and delivers a more durable, high-efficiency solution for circular saw guide pads. Finally, semi-industrial tests were performed on a full-scale guide pad to compare the wear resistance and failure behavior between the Babbitt alloy and the final Cu-Fe-based guide pad.

## Materials and methods

In this work, a Cu-5 vol.% NCG-6 vol.% CaF₂ composite, originally reported in Ref. 14 was modified by incorporating Fe powder (Rio Tinto, d₅₀ = 70 μm, purity ≥ 99.4%) in varying volume fractions ranging from 12 to 52 vol.%. The constituent powders were blended in a 2 L V-shaped mixer operating at 27 RPM for 20 min to ensure homogeneous mixing. The resulting mixtures were consolidated by uniaxial pressing at 490 MPa to produce green compacts. The selected compaction pressure was based on preliminary trials, which identified it as optimal for achieving maximum green density and structural integrity without inducing defects.

Sintering was carried out in a GSL1700X tube furnace at 1 010 °C for 60 min, using a controlled heating rate of 10 °C/min. To mitigate oxidation, the furnace chamber was continuously purged with high-purity nitrogen at a flow rate of 7.5 L/min. Following sintering, the specimens were cooled from 1 010 °C to 400 °C at an average rate of approximately 1.5 °C/s. Fabrication was performed in multiple discrete batches to ensure experimental reproducibility.

The chemical compositions of the prepared samples are listed in Table 1.

**Table 1:** Chemical composition of the composites

Sample	Fe (vol.%)	Cu- 5vol.%NCG—6vol.%CaF_2_
P0	0	Bal
P1	12	Bal
P2	22	Bal
P3	32	Bal
P4	42	Bal
P5	52	Bal

### Tribological testing

Tribological tests were carried out on a pin-on-disk tribometer (TRB3, Anton Paar) in accordance with ASTM G99 standards. A high-chromium steel ball with a diameter of 6 mm and a hardness of 60 HRC was used as the counterface material. Tests were performed under three different normal loads of 3 N, 6 N, and 10 N, while maintaining a constant sliding speed of 50 cm/s for a distance of 1 000 m. This sliding distance was selected in line with ASTM G99 recommendations to ensure sufficient test duration for the establishment of steady-state wear conditions, including friction stabilization and tribofilm development. Each composition was evaluated through a minimum of four replicate tests to ensure statistical reliability, and mean values were used for analysis. To quantify wear, the resulting wear tracks were analyzed using a Surtronic S-100 Series surface profilometer. Measurements were taken at five distinct locations along each wear track, and the average values were reported.

### Abrasive wear testing

Three-body abrasive wear behavior was assessed using a dry sand rubber wheel (DSRW, Falex NV-010) apparatus following the ASTM G65 standard (Procedure E). Silica sand with an AFS grain fineness of 50/70 served as the abrasive medium and was supplied at a constant feed rate of 330 g/min. Tests were performed under a normal load of 130 N with the rubber wheel rotating at 1 000 rpm. The density of the sintered composites was measured using the Archimedes method. The volume loss and the adjusted volume loss (accounting for the reduction in wheel diameter during testing) were calculated using the following expressions:

1 $$\mathrm{Volume}\;\mathrm{loss}, {\mathrm{mm}}^{3}=\frac{Mass loss(g)}{density(g/cm3)}\times 1000$$

2 $$\mathrm{AVL}\;=\;\mathrm{measured} \;\mathrm{volume}\; \mathrm{loss}\times \frac{228.6mm(9in.)}{wheel \;diameter \;after\; use}$$

### Mechanical tests

The hardness of the sintered materials was measured using a Brinell hardness (HBN) tester in accordance with ASTM E10-15. The test was conducted by applying a 500 kg load with a 10 mm diameter indenter and maintaining a dwell time of 15 s. Since the composites were made from powders with different hardness values and contain residual porosity, the reported values represent the apparent hardness rather than the intrinsic hardness of any single constituent. Each reported value corresponds to the average of at least 9 measurements (on 3 samples).

Standardized dog-bone composite specimens, prepared in accordance with MPIF Standard 10, were employed for tensile testing. The specimens were subjected to uniaxial tensile loading using a Satec T20000 testing machine, operated at a constant loading rate of 5 mm/min, until fracture occurred. The yield strength was determined from the engineering stress–strain curve using the 0.2% offset method. Three dog-bone specimens of Babbitt alloy were also prepared and tested to determine their tensile strength in relation to the sintered composites.

### Characterization

The microstructures of the composites were examined using a Keyence VHX-7000 digital microscope. Detailed microstructural observations of the composites and their worn surfaces were performed using a scanning electron microscope (SEM, FEI Inspect F50). Energy-dispersive spectroscopy (Ametek EDAX Octane Super-A) was used to provide a comprehensive understanding of the chemical composition of the tribo-layer generated on the worn surfaces. Additionally, the wear surface of the pin was thoroughly characterized using SEM to assess the nature and extent of wear. X-ray diffraction (XRD) was employed to identify the possible formation of new phases during the sintering process. The analysis was carried out in a *θ–2θ* configuration using Co Kα radiation (45 mA, 40 kV, λ = 0.1789 nm). Furthermore, a confocal optical microscope (ZEISS Smartproof 5) was used to analyze the three-dimensional surface morphology and topographical features of the composites, enabling detailed visualization of wear patterns, material loss, and surface roughness.

### Semi-industrial test

**Fig. 2:**
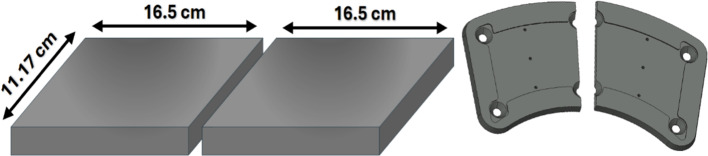
Schematic illustration of sintered composite blocks and the final guide pad geometry

**Fig. 3:**
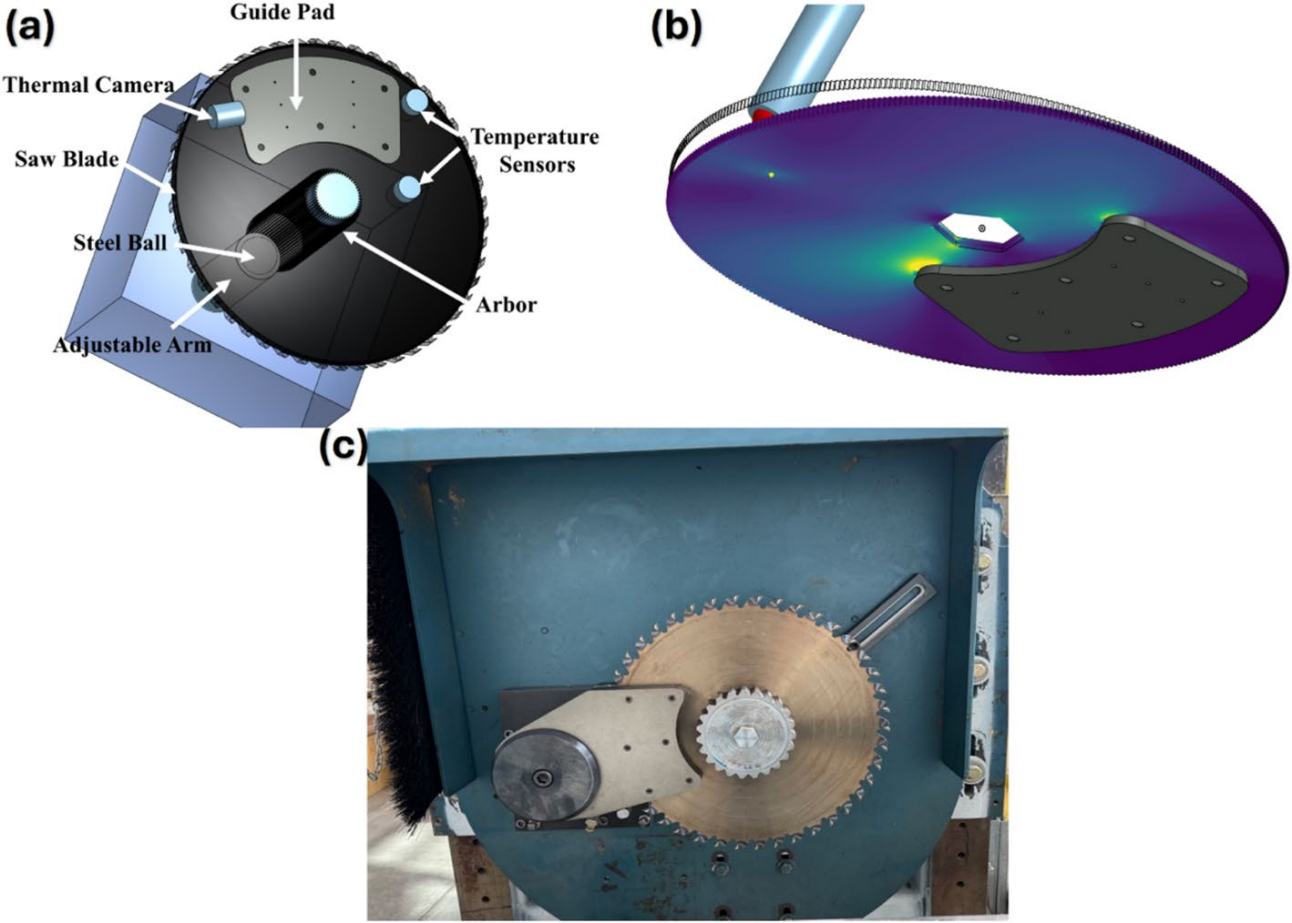
Schematic overview of the (**a**) saw system with integrated sensors, (**b**) articulated loading arm and (**c**) the complete test bench setup

In order to test and evaluate the performance of the full-scale composite guide pad, the optimized Cu-Fe-based composite premix was sent to Powder Tech Associates (North Andover, MA, USA) for compaction and sintering. Owing to the substantial dimensions of the full-sized guide pad, the powder was compacted and sintered in two separate blocks (16.5 cm × 11.17 cm × 0.75 cm), which were subsequently precision-machined at AmecPro (Lévis, QC, Canada) to the final guide-pad geometry (Fig. 2). The machined guide pads were then delivered to the Clermond Hamel sawmill (Québec, Qc, Canada) for surface finishing and final rectification to obtain a smooth, uniform contact surface before testing. The experimental tests were performed under simulated operating conditions at the Faculty of Forestry, Geography and Geomatics, Gene-H.-Kruger Building, Laval University (Québec, Qc), to evaluate and compare the performance of the Cu-Fe composite against that of the conventional Babbitt material. To simulate the pressure exerted (by wood/or saw blade) on the guide pad within a sawmill setting, an articulated arm equipped with a steel ball at one end (to minimize friction between the arm and the saw blade during operation) was developed (Fig. 3). This arm was designed to be affixed to the rear of the saw blade on the test bench. By manipulating the length of this adjustable arm, pressure was intentionally applied to the saw blade, thereby indirectly exerting force on the guide pad.

The arm functions as a mechanical lever, where increasing the arm length generates a higher bending moment on the blade, resulting in greater deflection and increased contact pressure at the guide pad interface. This approach enables a controlled transition from low to severe contact conditions, allowing systematic evaluation of the composite material response under varying mechanical stresses. Furthermore, the gradual increase in arm length ensures stable loading conditions and minimizes sudden impact effects, thereby improving the repeatability and reliability of the experimental results. The arm was positioned at the midpoint of the saw radius to ensure that the indirect pressure exerted on the guide pad is evenly distributed. Two temperature sensors were positioned under the guide pad, located in close proximity to both the rim and the eye of the saw, for temperature measurement purposes. Additionally, a Micro-Epsilon thermoMETER TIM 8 High resolution thermal (infrared) camera was mounted at the top center of the guide pad to measure the surface temperatures of the Babbitt and composite guide pads during operation. Throughout the test, the power consumption of the test bench saw was also recorded for subsequent analysis.

The test sequence commenced with the arm length set to 0 mm, allowing the saw blade to rotate freely and establish a baseline for measuring power consumption and temperature of the guide pad. The blade was operated at 2 700 RPM to replicate standard sawmill operational speed for a duration of five minutes. In each successive run, the arm length was incrementally extended by 2 mm to gradually increase the applied load on the guide pad. Before each test, approximately 7–10 ml of liquid lubricant (Hipertech Synthetic Oil, SAE 5W-40 grade) was applied to the saw blade surface. After every test, both the saw blade and guide pad were allowed to cool naturally to room temperature. This procedure was repeated until the arm length reached 8 mm, beyond which the saw rim, saw eye, and the guide pad temperatures rose sharply for both the Babbitt and composite guide pads.

## Results and discussions

**Fig. 4:**
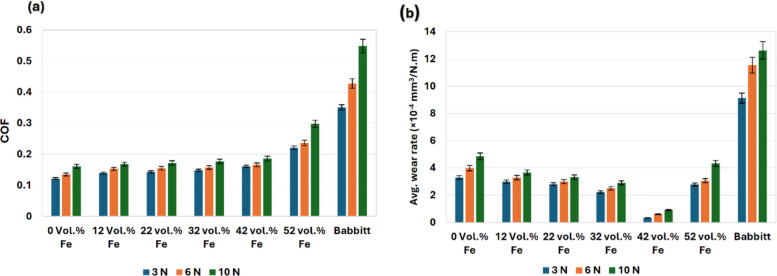
The effect of Fe on (**a**) COF and (**b**) wear rate of Cu-Fe-based composites (Measurements were obtained with the pin-on-disk test)

Figure 4 illustrates the variation in the COF and wear rate for pin-on-disk wear test under applied loads of 3 N, 6 N and 10 N for composites containing different Fe volume fractions, ranging from 12 vol.% to 52 vol.%. Additionally, the COF and wear rate of Cu-5vol%−6vo l%CaF_2_ composite (composite without Fe, P0) and Babbitt are provided for comparison.

The incorporation of 12 vol.% Fe (P1) leads to an increase in the COF across all applied loads, showing relative rises of 13.9% (for 3 N), 13.3% (for 6 N), and 4.3% (for 10 N) compared with the Fe-free composite. A clear trend is also observed in all composites in which the COF increases with increasing applied load. However, due to the balanced interaction between Fe and the Cu matrix, only a slight and gradual increase in COF is observed among the composites containing 12–42 vol.% Fe (P1–P4) under each load condition. Within this range, the Cu matrix remains the dominant factor in the composite. The uniform dispersion of Fe particles within this range leads to a homogeneous microstructure, ensuring even load distribution and minimizing localized stress concentrations during sliding (Zhang et al. 2024).

The wear rate of the composites (Fig. 4b) also demonstrates a clear upward trend with increasing applied load, which is attributed to the elevated real contact pressure at the asperity contacts between the pin and surface. This effect is most pronounced in the Fe-free composite, where the absence of reinforcing particles leaves the relatively soft Cu matrix prone to significant material removal and deformation. As a result, the wear rate in P0 composite increases by approximately 46% with rising load up to 10 N, accelerating material removal and leading to severe wear rate. The coefficient of friction (COF) and wear rate do not exhibit a direct correlation, as they are governed by different mechanisms. Wear is mainly controlled by material removal and plastic deformation, whereas friction depends on interfacial shear strength and the nature of the contact layer (Lontin and Khan 2021; Zhu and Li 2022). In this study, increasing Fe content up to 42 vol.% (P4) significantly reduces wear rate, while only a slight increase in COF is observed. This higher wear resistance is attributed to the improved hardness and load-bearing capacity imparted by Fe, which suppresses plastic deformation and enhances wear resistance. Previous studies on Cu–Fe tribological systems have shown that, under low sliding speed conditions, increasing the Fe content in the third body can increase the coefficient of friction (COF) due to the higher shear resistance and lower plastic accommodation capability of Fe-rich tribolayers compared with Cu-rich transfer films, which facilitate smoother interfacial sliding (Xiang et al. 2005). Fe addition also reduces the relative contribution of lubricating phases, resulting in a modest rise in COF. Nevertheless, the continuity of the Cu matrix up to 42 vol.% Fe limits this increase, maintaining stable friction behavior. Therefore, the observed behavior does not represent an anomaly, but rather highlights that in such composites, wear resistance is governed more strongly by mechanical integrity and load-bearing capability than by friction alone.

**Fig. 5:**
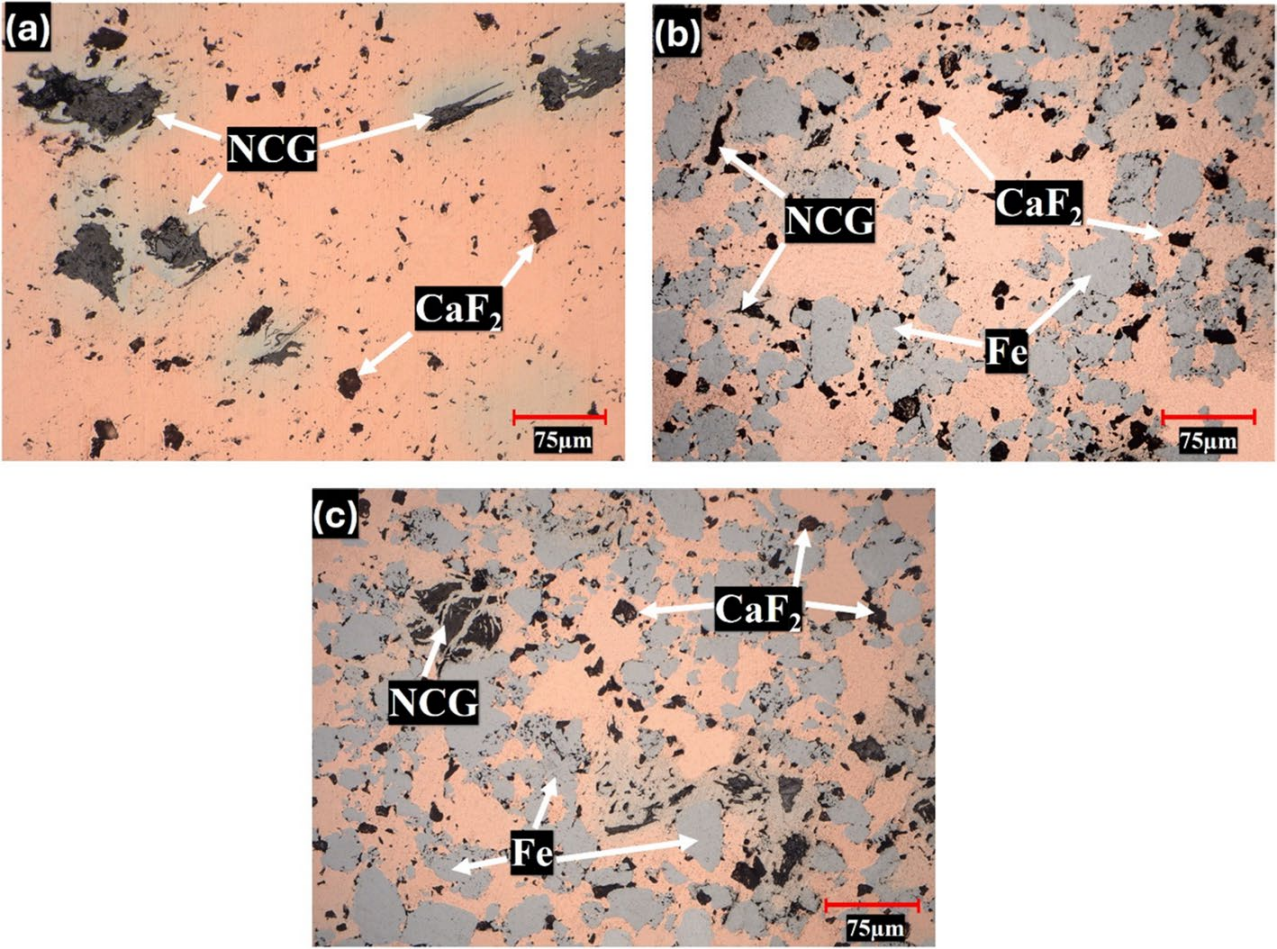
Digital micrographs of (**a**) P0, (**b**) P4 and (**c**) P5 composites

Figure 5 presents digital micrographs of the P0, P4, and P5 composites, illustrating the influence of increasing Fe content on microstructural architecture. The Fe-free composite (P0) exhibits a continuous Cu matrix with a homogeneous dispersion of NCG and CaF₂ particles. In the P4 composite (42 vol.% Fe), Fe particles form an interconnected reinforcing network, however, the Cu matrix remains continuous, reflecting a balanced matrix–reinforcement configuration in which Fe acts primarily as a strengthening phase rather than a structural matrix replacement. In contrast, the P5 composite (52 vol.% Fe) displays a structural transition, where Fe becomes the dominant phase and the Cu matrix is largely confined to discontinuous regions between Fe particles indicating a reinforcement-dominated microstructure. Despite this shift in phase dominance, both NCG and CaF₂ remain well dispersed within the Cu-rich regions across P4 and P5 composites.

**Fig. 6:**
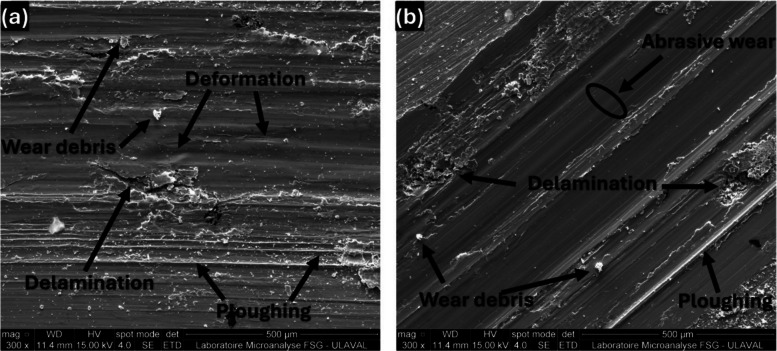
SEM micrographs of the worn surface of P0 (Fe-free) composite under 10 N load, indicating (**a**) delamination, deformation, (**b**) abrasive wear and ploughing

The SEM micrographs in Fig. 6 reveals extensive material loss and ploughing across the wear track of the Fe-free composite under the 10 N load, confirming its limited resistance to high-stress sliding conditions.

**Fig. 7:**
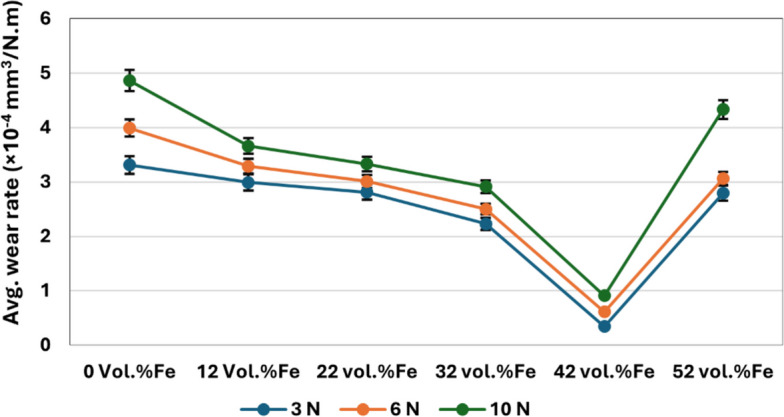
The effect of load and Fe addition on the wear rate of the Cu-Fe-based composites

On the other hand, a steady reduction in wear rate is observed across all applied loads within the Fe content range of 12–42 vol.% (P1–P4). As illustrated in Fig. 7, this interval clearly demonstrates the beneficial influence of Fe incorporation, with wear resistance progressively improving as the Fe fraction increases. The enhanced wear resistance arises from the strengthening role of Fe particles within the matrix. Fe acts as a mechanically robust reinforcing phase that shares an increasing of the applied load as the Cu matrix begins to deform, thereby reducing the effective stress within the matrix (Zhang et al. 2021; Yu et al. 2021). Table 2 summarizes the percentage reduction in wear compared to Babbitt for all composites under the three applied load conditions. Notably, the composite containing 42 vol.% Fe (P4) exhibited the greatest improvement, demonstrating an approximate 94% reduction in wear relative to Babbitt across all load levels.

**Table 2:** Percentage reduction in wear of Cu-Fe composites compared to Babbitt under different applied loads

Load (N)	P0 (%)	P1 (%)	P2 (%)	P3 (%)	P4 (%)	P5 (%)
3	63.70	67.21	69.18	75.54	96.27	69.40
6	65.42	71.49	73.91	78.33	94.71	73.48
10	61.52	71.02	73.63	76.95	92.79	65.71

The reinforcement provided by Fe helps avoid the initiation of wear and minimizes the impact of adhesive and abrasive wear mechanisms (Zhou et al. 2019). A balance between Fe and Cu results in strong adhesion, driven by enhanced wettability and slight solubility between the two materials (Xiong et al. 2007). This optimal Fe content strengthens the matrix, improves the mechanical properties of the friction surface, and effectively minimizes wear loss.

**Fig. 8:**
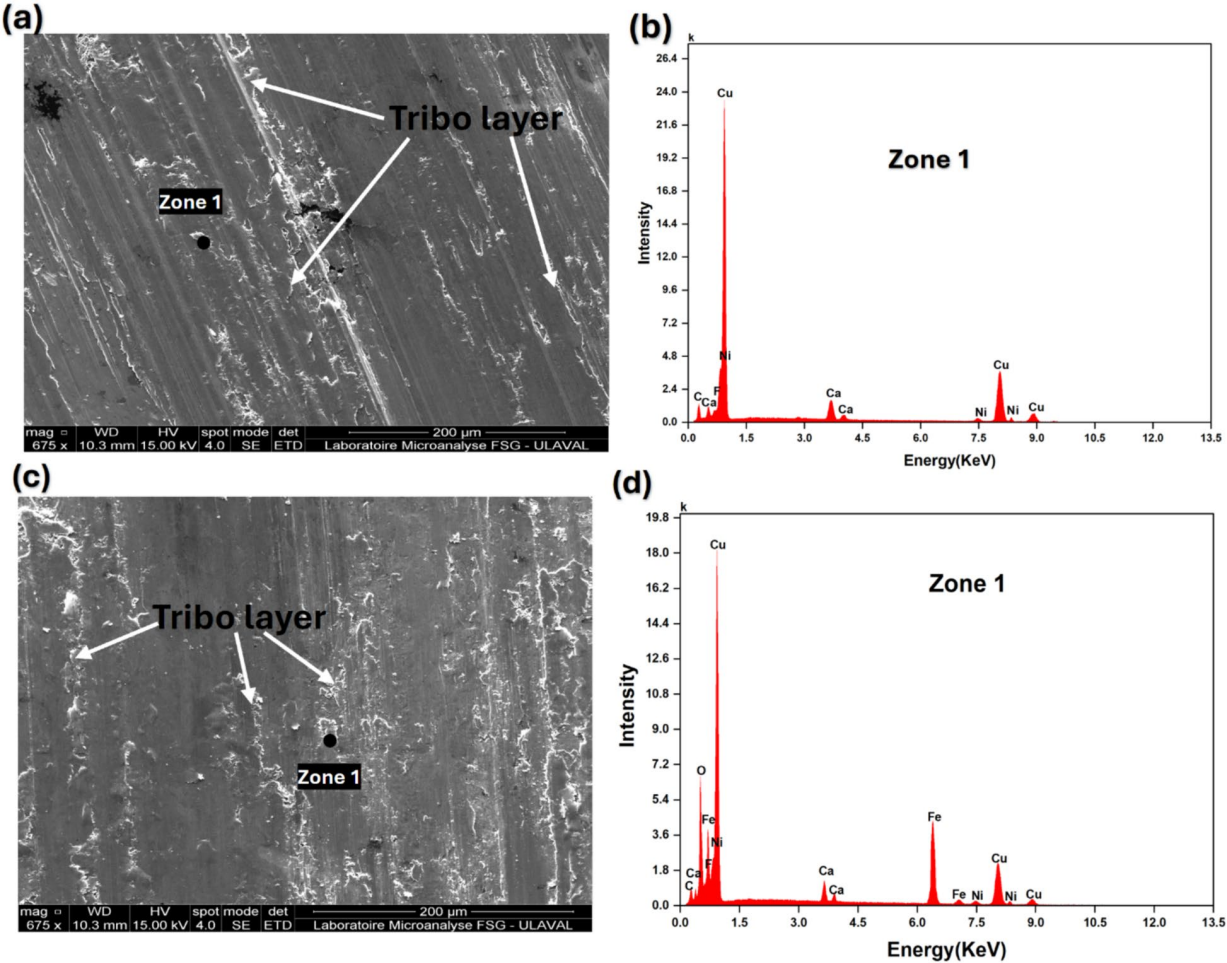
**a**, **b** SEM/EDS characterization of the tribo layer formed on the wear track of the P0 and **c**, **d** P4 composites under 6 N load

An increased Fe content promotes the formation of a protective tribo-oxide layer on the contact surface, owing to the higher oxidation propensity of Fe compared to Cu (Peng et al. 2018) during pin-on-disk-test. This tribo-oxide layer acts as a barrier, preventing metal-to-metal contact and protecting the underlying substrate from damage. Figure 8 presents the SEM and EDS analyses of the surface on the wear tracks of the Fe-free composite (P0) and the Fe-reinforced composite containing 42 vol.% Fe (P4). In the absence of Fe, the worn surface of P0 shows no evidence of tribo-oxide formation, indicating limited oxidative activity during sliding (Fig. 8b). In contrast, the P4 specimen exhibits a pronounced enrichment in oxygen together with other alloying elements, pointing to the development of a well-established tribo-oxide layer (Fig. 8d). This oxide-rich tribo-layer stabilizes friction and wear by forming a protective film that regulates oxide retention on the worn surface and limits direct metal-to-metal contact, thereby reducing wear severity and enhancing the overall wear resistance of the composite.

On the other hand, beyond 42 vol.% Fe, the COF and wear rate sharply increase (Fig. 7) in all applied loads due to the oversaturation of Fe, which leads to the formation of Fe-rich phases. This over-saturation reduces the composite's ductility and toughness, making it more prone to fracture under sliding conditions.

**Fig. 9:**
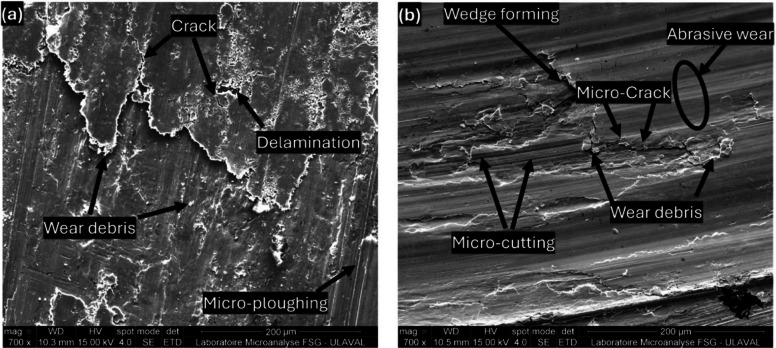
SEM micrographs of the worn surface of the composite containing 52 vol.% Fe under a 6 N load showing (**a**) cracks and delamination, and **b** wedge formation and abrasive wear

In the SEM micrographs presented in Fig. 9, the wear track on the P5 composite material surface exhibits several distinctive features that reveal the underlying wear mechanisms. The presence of cracks propagating through the material indicates progressive fracture, leading to fragmentation and the subsequent formation of wear debris. The irregular, jagged edges alongside flake-like regions suggest that material removal occurs in layers due to subsurface cracking and interfacial weakening. At this Fe content, the excessive amount of Fe at the surface promotes the formation of thick, brittle, and poorly adherent tribo layers. Rather than stabilizing the COF, these tribo layers tend to spall under stress, producing abrasive particles that accelerate material loss. In parallel, the harder Fe particles exposed at the worn surface increase roughness and intensify asperity interactions (Xiong et al. 2007) (Fig. 4a).

**Fig. 10:**
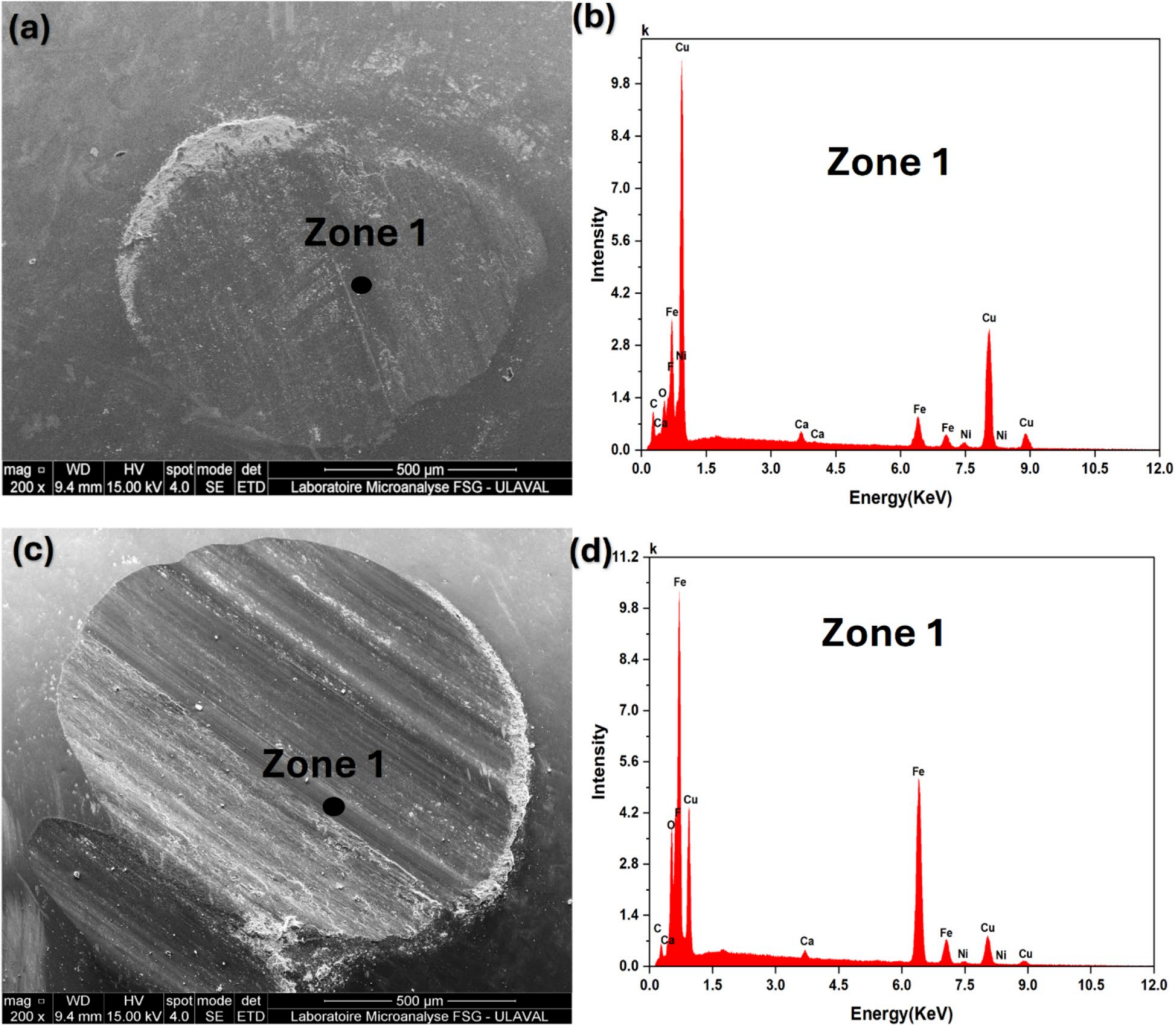
SEM micrographs (**a**,**c**) and corresponding EDS spectra (**b**,**d**) of worn steel balls after pin-on-disk testing against P4 and P5 composites at 50 cm/s under a 6 N load

Figure 10 presents the SEM micrographs and corresponding EDS spectra of the steel balls tested against composites containing 42 vol.% Fe (P4) and 52 vol.% Fe (P5) at a sliding speed of 50 cm/s under a normal load of 6 N. For the P4 composite, the EDS spectrum is dominated by a strong Cu signal (Fig. 10b), indicating that the transfer layer formed on the steel ball surface primarily consists of Cu-rich material originating from the P4 composite. In contrast, the steel ball tested against the P5 composite exhibits pronounced Fe and O signals (Fig. 10d), confirming substantial transfer of Fe-rich material and the presence of Fe-based oxides on the counterface. Notably, the absence of detectable Cr peaks indicates that the observed Fe signal does not originate from the steel counterface, but rather from Fe-rich debris transferred from the tribo layer. This Fe-rich transfer reflects the formation of an unstable tribo-oxide layer that undergoes repeated disruption during sliding, facilitating debris generation and enhancing abrasive interactions. Consequently, the elevated Fe signal detected by EDS correlates with the reduced tribological stability, higher friction coefficient, and increased wear observed at the highest Fe (52vol.%) content.

**Fig. 11:**
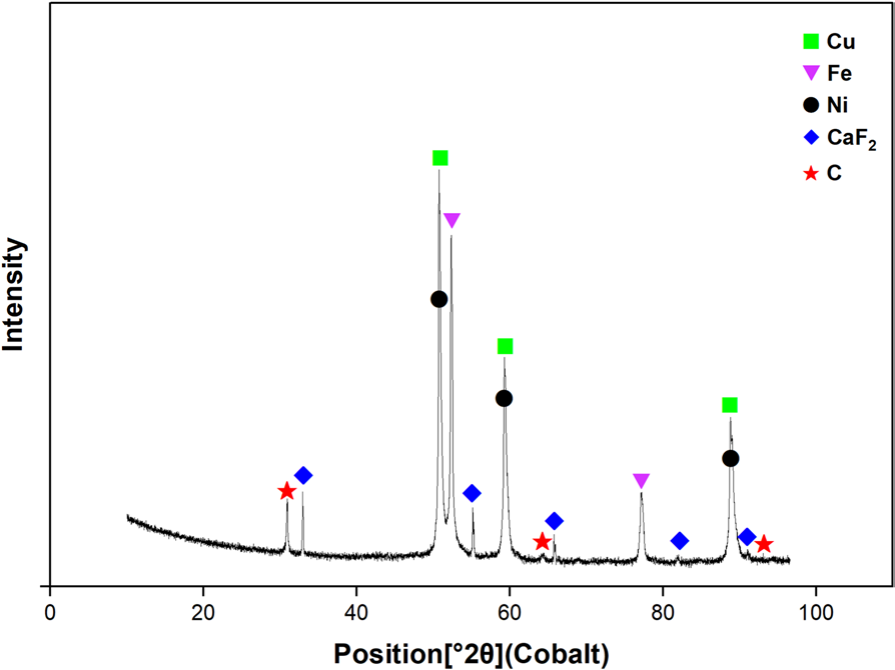
XRD spectrum obtained from P4 composite

The XRD analysis of the P4 sample (42 vol.% Fe) reveals that all diffraction peaks correspond exclusively to the initial constituents, indicating that no secondary or reaction-induced phases were formed during sintering (Fig. 11). This observation confirms that the composite system remained chemically stable under the applied processing conditions. CaF₂ is well known for its excellent chemical inertness and high thermal stability, and it does not undergo decomposition or chemical interaction with the metallic matrix during high-temperature processing (Liu et al. 2021).

Similarly, the stability of iron within the composite is governed by its limited solubility in copper and the absence of stable Cu–Fe intermetallic compounds under equilibrium conditions. According to established thermodynamic assessments and phase diagram studies of the Cu–Fe system, the two elements exhibit negligible mutual solubility in the solid state and a strong tendency toward phase separation. As a result, Fe is retained as a distinct phase within the Cu matrix rather than participating in the formation of new compounds (Lindqvist and Uhrenius 1980).

**Fig. 12:**
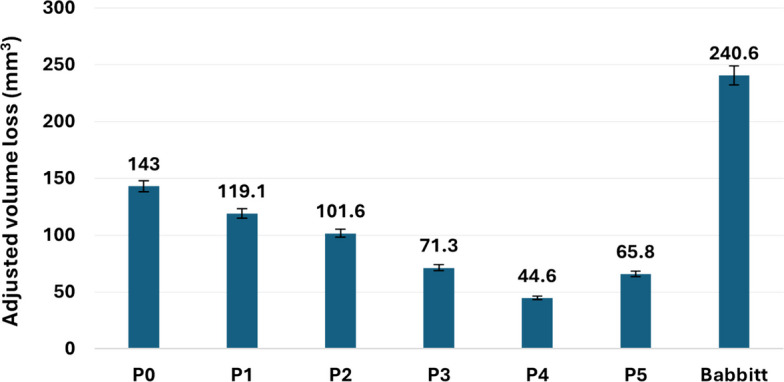
DSRW abrasion test result for composites with varying Fe content

Figure 12 illustrates the volume loss results from the DSRW abrasion test, which are consistent with the trends observed in the pin-on-disk experiments. As summarized in Table 3, the incorporation of Fe progressively reduced the volume loss of the composites relative to Babbitt, with the minimum wear observed for the specimen containing 42 vol.% Fe (P4). At this composition, the composite demonstrated the greatest abrasion resistance, with a nearly 81% reduction in volume loss relative to Babbitt. Beyond this optimal level, however, the resistance to abrasion declined. This improvement up to the optimal Fe content is attributed to enhanced hardness and the development of a balanced microstructure that promotes efficient load distribution while preserving the toughness of the composite.

**Table 3:** Percentage reduction in volume loss of all composites relative to Babbitt during DSRW

P0 (%)	P1 (%)	P2 (%)	P3 (%)	P4 (%)	P5 (%)
40.56	50.49	57.77	70.36	81.46	72.65

At higher reinforcement levels (52 vol.% Fe), the composite microstructure becomes less homogeneous, with excessive Fe-rich regions acting as preferential sites for crack initiation and propagation under abrasive loading (as shown in SEM micrograph in Fig. 9). This, results in a loss of toughness and a reduced ability of the composite to accommodate localized stress (Greasley and Shi 1993; Llorca and Gonzalez 1998; Segurado and LLorca 2006). Moreover, the reduced Cu matrix fraction above 42 vol.% Fe limits the ductile phase needed to provide adequate support for the hard phases. Consequently, the composite exhibits higher brittleness and a diminished capacity for energy absorption, which collectively contribute to the decrease in abrasion resistance beyond the optimal Fe content (Minitsky et al. 2019).

**Fig. 13:**
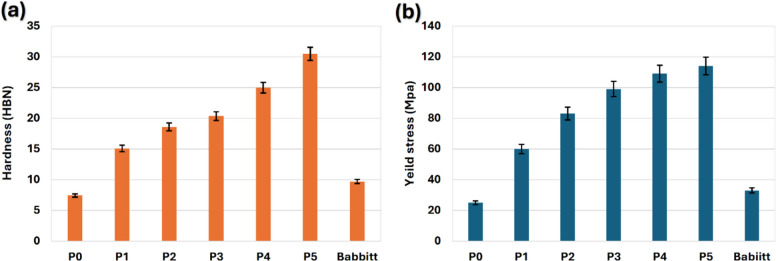
(**a**) Brinell Hardness and (**b**) Yield Strength of composites with varying Fe content

Figure 13 presents the variation in hardness and yield stress of Cu-Fe-based composites containing different Fe contents relative to Babbitt. The data clearly shows a steady improvement in mechanical properties as the Fe content increases. As shown in Fig. 13a hardness rises progressively, highlighting the strengthening role of Fe. Generally harder materials typically exhibit lower wear rates owing to their greater resistance to surface deformation and material removal (Mokhtar 1982; Sami et al. 2022; Hegde et al. 2024). Similarly, the yield stress in Fig. 13b demonstrates a similar trend, with the composite containing 42 vol.% Fe achieving a value approximately three times greater than that of Babbitt. The uniformly dispersed Fe particles within the Cu matrix contribute to load-transfer strengthening, as the harder Fe regions absorb a larger portion of the applied stress and restrict plastic deformation in the softer Cu phase (Maurya et al. 2022; Zhang et al. 2022).

**Fig. 14:**
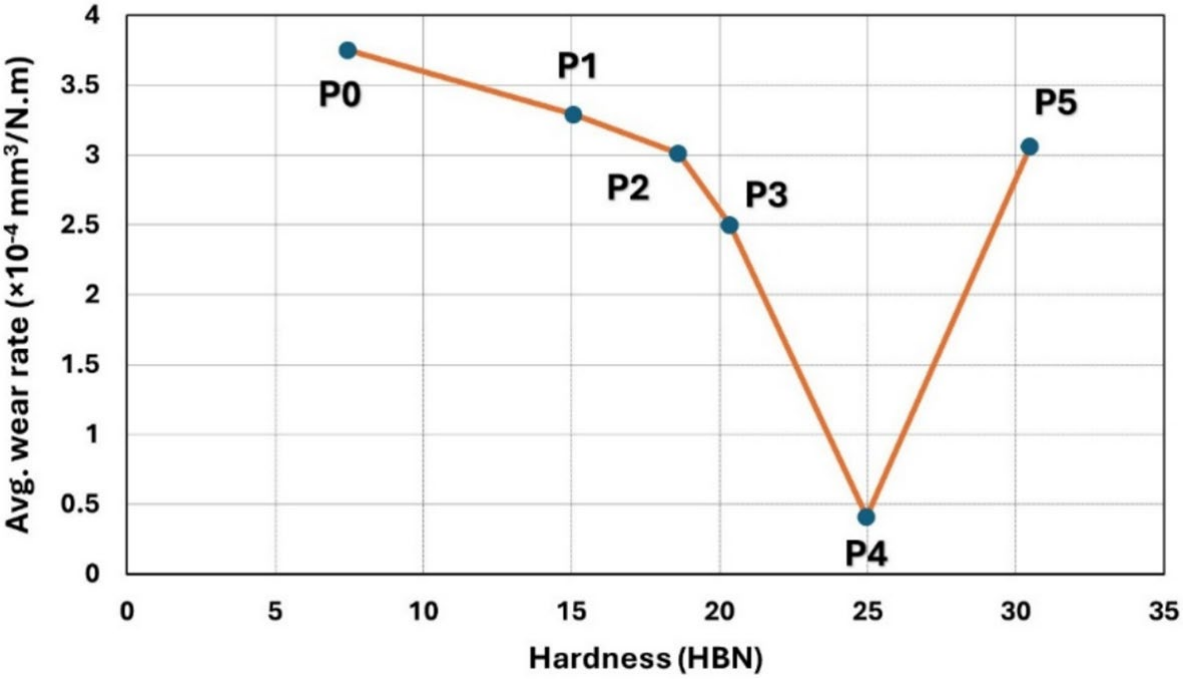
Wear rate as a function of Brinell hardness

The relationship between average wear rate and Brinell hardness for Cu-based composites (P0 – P5) is presented in Fig. 14. According to Archard’s classical wear equation $(V=KWL/H)$, the wear rate (V) is expected to vary inversely with hardness (H). Up to the P4 composite, the observed trend follows this theoretical prediction, with increasing hardness leading to a pronounced reduction in wear rate. This behaviour reflects the beneficial influence of higher hardness in suppressing asperity deformation and material removal under sliding contact, thereby enhancing wear resistance. Nevertheless, the results reveal a notable deviation from Archard’s prediction for the P5 specimen. Despite exhibiting the highest hardness, P5 shows a significant increase in wear rate relative to P4. This discrepancy indicates that the wear process in such multiphase composites cannot be adequately described by hardness alone. These findings underscore the limitations of purely hardness-based predictive models and highlight the importance of incorporating microstructural, interfacial mechanisms considerations when evaluating the wear mechanisms of multiphase composites containing solid lubricants and reinforcing phases.

This deviation from Archard’s assumption arises because the model presumes uniform and purely plastic contact behaviour, which is not valid for heterogeneous and multiphase materials. In such composites, wear is governed not only by hardness but also by complex interactions among different microstructural constituents. Factors such as differential deformation between the hard and soft phases, localized delamination, third-body film formation, and the intermittent exposure of lubricating particles significantly modify the real contact area and frictional response. As a result, the overall wear mechanism becomes more dependent on the microstructural integrity and interfacial stability than on hardness alone.

In the case of the P5 composite (52 vol.% Fe), the microstructure becomes increasingly Fe-dominated, with a reduced and discontinuous Cu matrix. This limits the material’s ability to accommodate localized stresses through plastic deformation, leading to stress concentration at Fe–Cu interfaces and within Fe-rich regions. Consequently, crack initiation and propagation are promoted under sliding conditions, resulting in subsurface damage and delamination (As shown in SEM micrograph in Fig. 9). In addition, the excessive Fe content favors the formation of thick, brittle, and poorly adherent tribo-oxide layers. Unlike the stable and protective tribo-layer observed in the P4 composite, these oxide layers are prone to repeated fracture, generating third-body debris. These debris particles act as abrasive elements, intensifying micro-cutting and ploughing at the contact interface. As a result, the dominant wear mechanism shifts from mild abrasive/adhesive wear to severe abrasive and delamination-driven wear, leading to increased material loss despite the higher hardness of the P5 composite. Similar deviations from Archard-type behaviour in multiphase and particle-reinforced systems have been reported in the literature, where microstructural heterogeneity and unstable tribo-layers govern wear response rather than hardness alone (Hu et al. 2022).

### Semi-industrial test

**Fig. 15:**
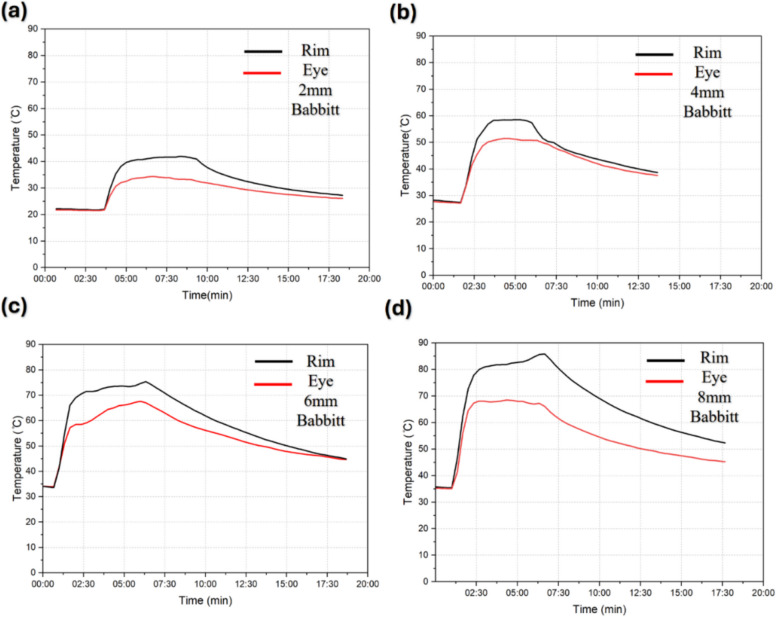
Saw rim and eye temperature measurements for the Babbitt guide pad at arm lengths of (**a**) 2 mm, (**b**) 4 mm, (**c**) 6 mm, and (d) 8 mm

**Fig. 16:**
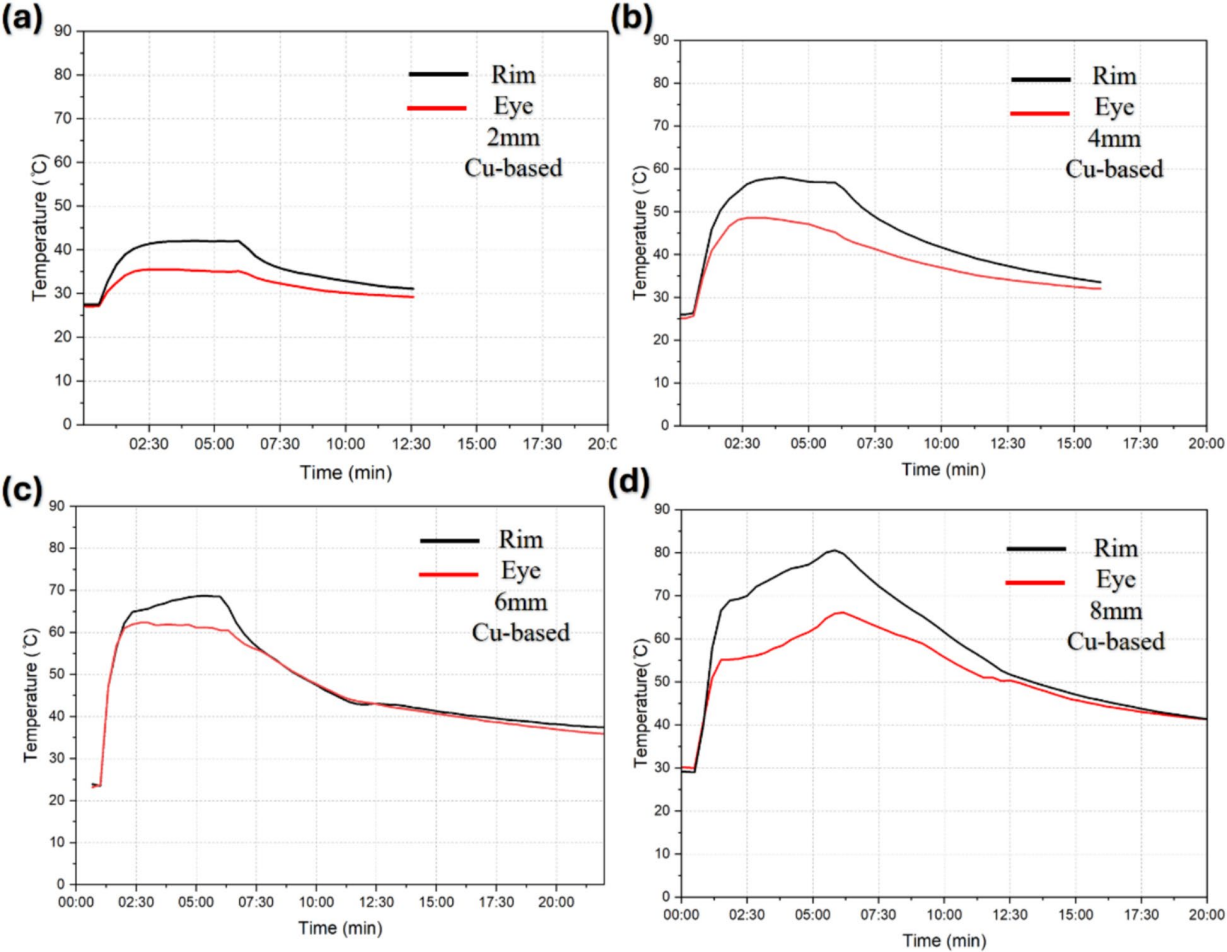
Saw rim and eye temperature measurements for the Cu-based composite guide pad at arm lengths of (**a**) 2 mm, (**b**) 4 mm, (**c**) 6 mm, and **d** 8 mm

Figures 15 and 16 present the temperature evolution at the saw rim and eye when Babbitt and composite guide pads used under varying deflection conditions. For both materials, increasing the arm length, which corresponds to a higher deflection angle, led to a gradual and consistent rise in temperature at both measurement points (rim and eye). In all cases, the rim exhibited a higher temperature than the eye, consistent with industrial observations. Table 4 summarizes the maximum temperatures recorded at the rim and eye for the Babbitt and composite guide pad, clearly showing that the use of a Babbitt guide pad leads to a higher temperature elevation at both positions, especially at higher deflections. This response is attributed to the intrinsically lower thermal conductivity of Babbitt, which limits its ability to dissipate frictional heat efficiently, thereby allowing heat to accumulate at the contact interface resulting in elevated temperatures on the saw blade. In contrast, the composite guide pad demonstrates reduced peak temperatures consistent with the superior heat-spreading capabilities of the Cu matrix.

**Table 4:** Peak temperature values at Rim and Eye locations for Babbitt and Cu-Fe-Based guide pads

	2 mm		4 mm		6 mm		8 mm	
	Eye(˚C)	Rim(˚C)	Eye(˚C)	Rim(˚C)	Eye(˚C)	Rim(˚C)	Eye(˚C)	Rim(˚C)
Babbitt	34.35	41.95	51.50	58.49	67.59	75.37	68.51	85.86
Cu-based	35.05	42.04	48.62	58.01	61.84	67.90	65.31	80.07

**Fig. 17:**
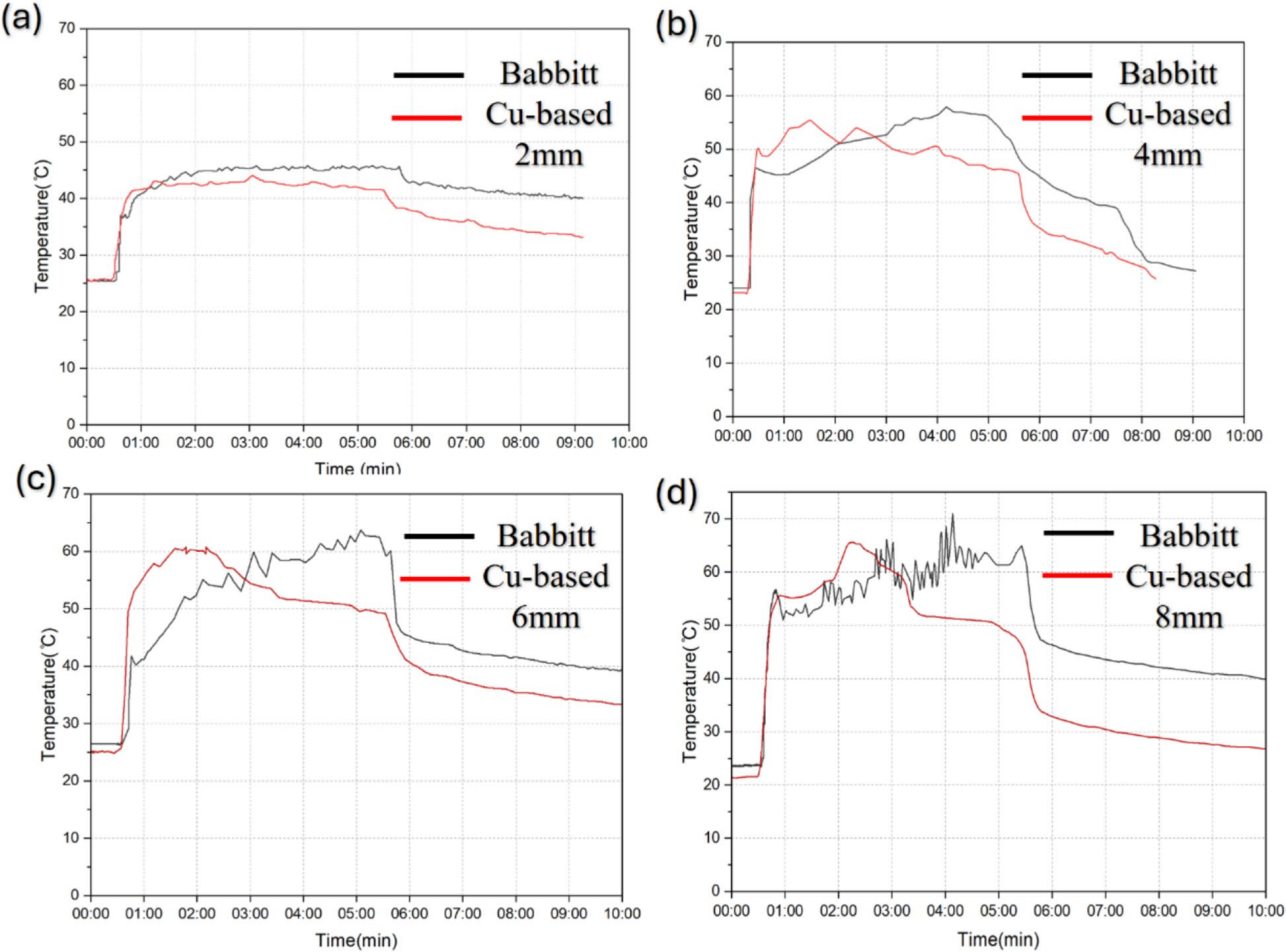
Temperature evolution of both guide pads during successive test runs at arm lengths of (**a**) 2 mm, (**b**) 4 mm, (**c**) 6 mm, and **d** 8 mm

Figure 17 compares the temperature evolution of the Babbitt and composite guide pads during successive tests at different deflection angles recorded by a thermal camera. As observed, both guide pads exhibit an initial sharp temperature increase at the onset of sliding, due to frictional heating. However, their subsequent thermal responses differ markedly. In the case of a composite guide pad, the temperature gradually decreases and stabilizes with continued sliding, signifying the attainment of a steady state tribological regime and effective thermal stabilization. The high thermal conductivity of the Cu matrix allows efficient heat dissipation through the bulk of the pad, facilitating thermal equilibrium and preventing temperature accumulation at the contact zone. This response highlights the composite’s ability to effectively transfer heat away from the contact zone, thereby minimizing localized overheating during sliding. In contrast, the Babbitt guide pad exhibits a continuous temperature rise throughout the test duration, with no stabilization before the test ends. This persistent increase reflects the alloy’s limited thermal conductivity. As a result, frictional heating accumulates faster than it can be dissipated, leading to localized softening of the Babbitt surface and increased adhesive interactions with the saw blade. Elevated temperature, coupled with high contact pressure, significantly increases the likelihood of plastic deformation in Babbitt guide pads during operation. This is primarily due to the reduction in yield strength of Babbitt at elevated temperatures, which promotes localized softening and plastic flow under load.

**Fig. 18:**
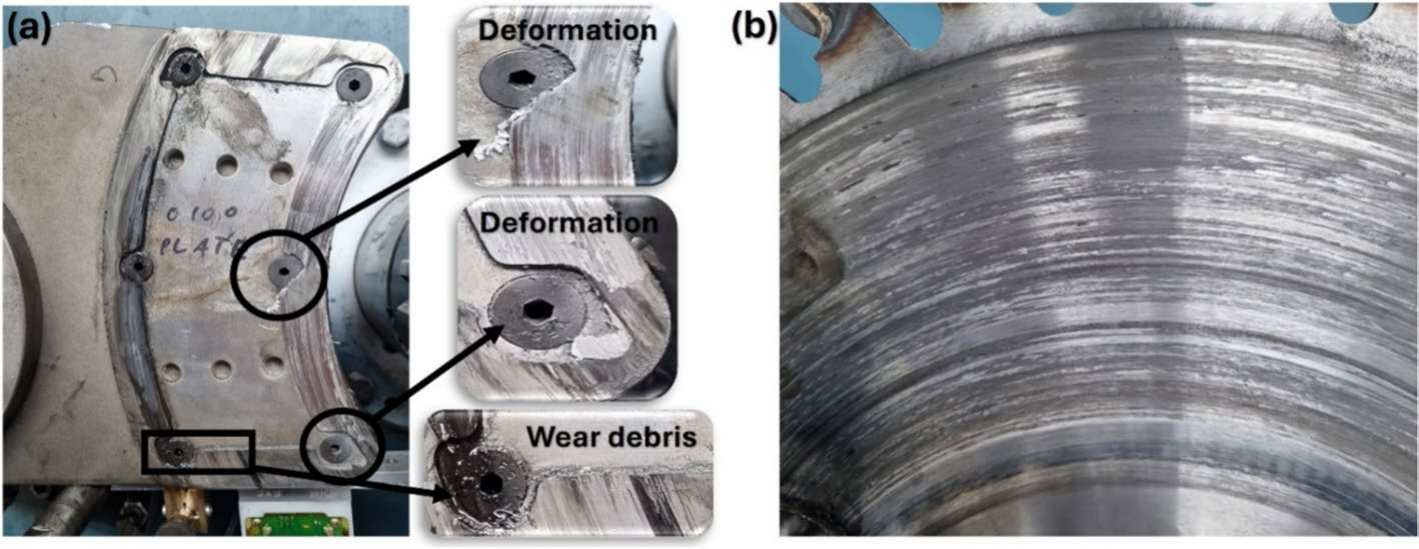
(**a**) Babbitt guide pad surface and (**b**) saw surface after the test

**Fig. 19:**
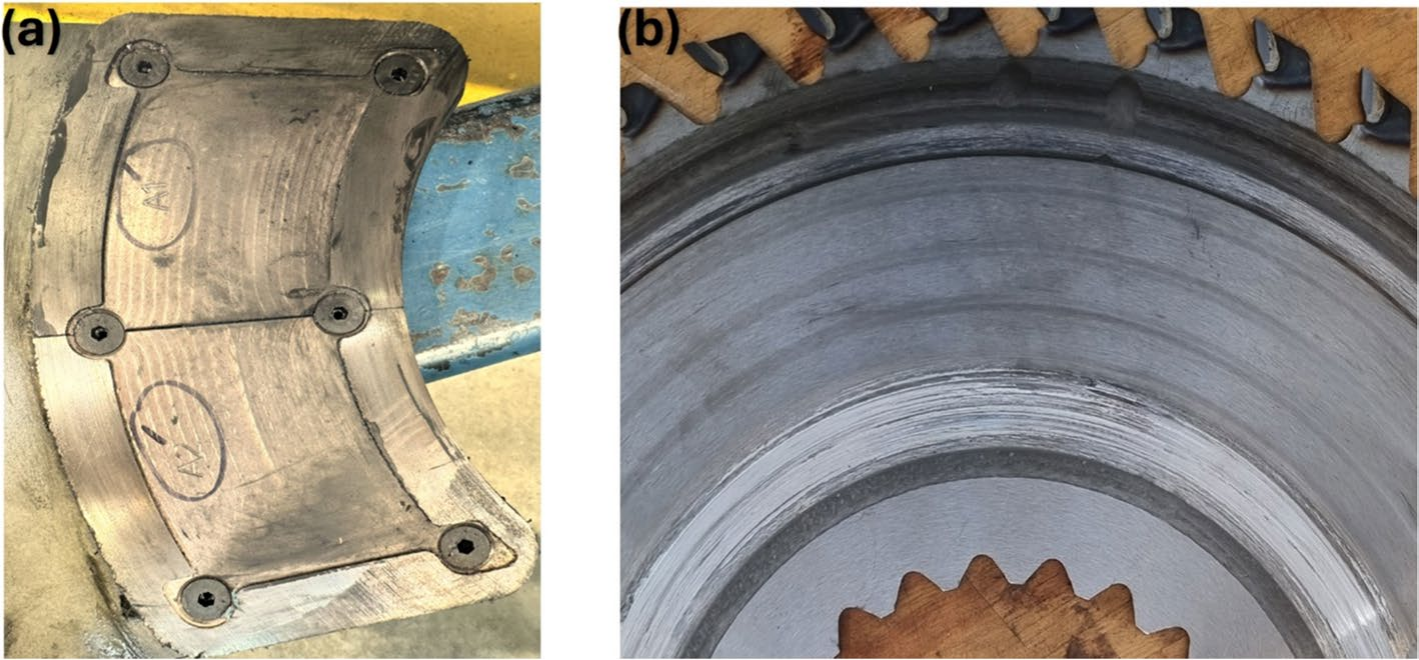
(**a**) Cu-Fe-based guide pad surface and (**b**) saw surface after the test

**Fig. 20:**
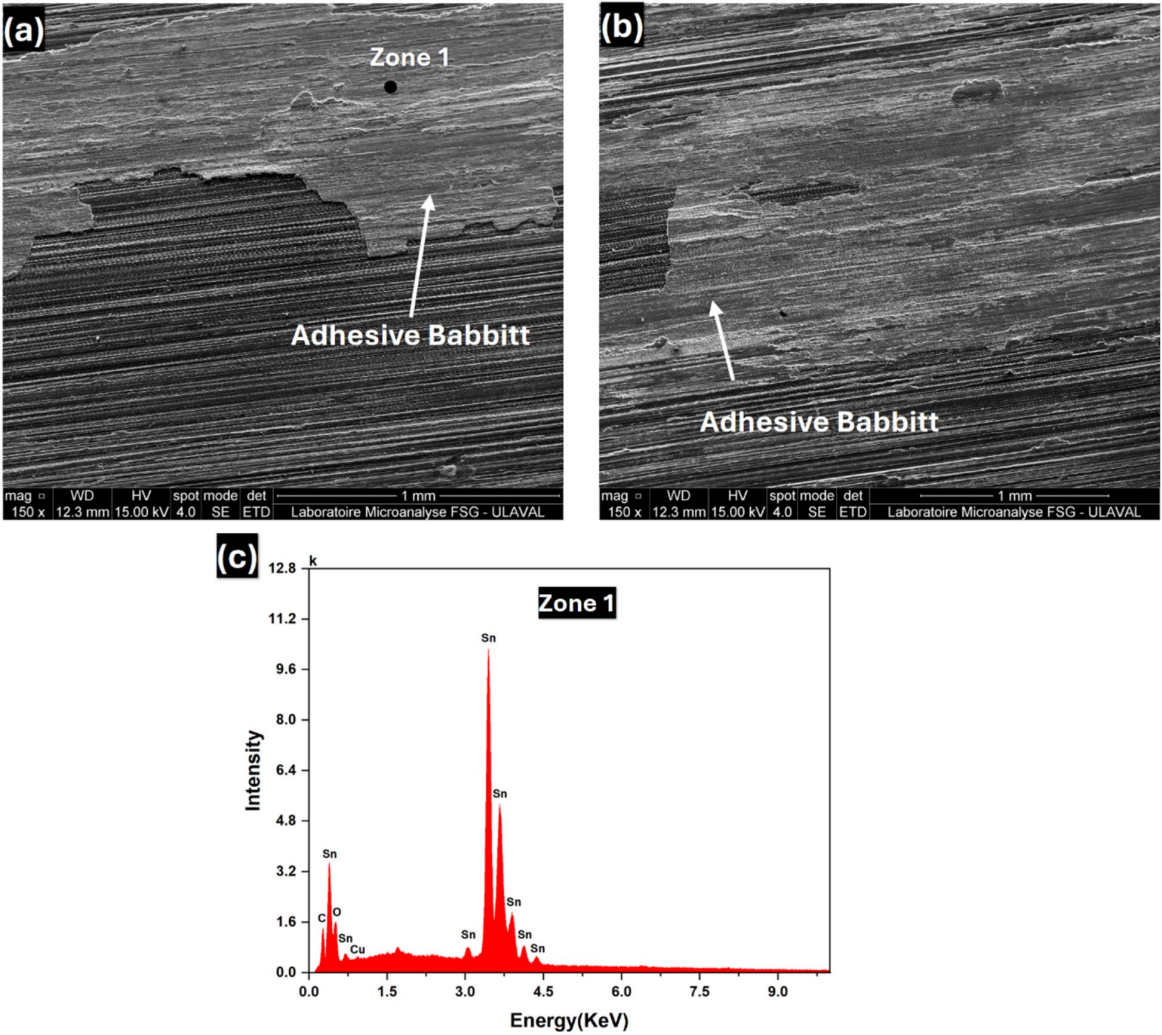
(**a**,**b**) SEM micrographs and (**c**) corresponding EDS analysis of the saw surface indicating extensive adhesion of Babbitt during sliding

Figures 18 and 19 show the contact surfaces of the Babbitt and composite guide pads after the semi-industrial test, along with their corresponding saw surfaces. As observed, the Babbitt guide pad experienced extensive deformation and a considerable accumulation of wear debris, particularly along the edges, indicating a high wear rate. The saw surface in contact with the Babbitt also exhibits noticeable adhesive transfer of Babbitt material, confirming the occurrence of severe adhesive wear. SEM/EDS analyses of the saw surface further confirm the strong adhesion of Babbitt material to the saw blade, verifying the occurrence of intense adhesive interactions during sliding (Fig. 20). In contrast, the composite guide pad displays negligible surface damage, with no visible material transfer to the saw.

**Fig. 21:**
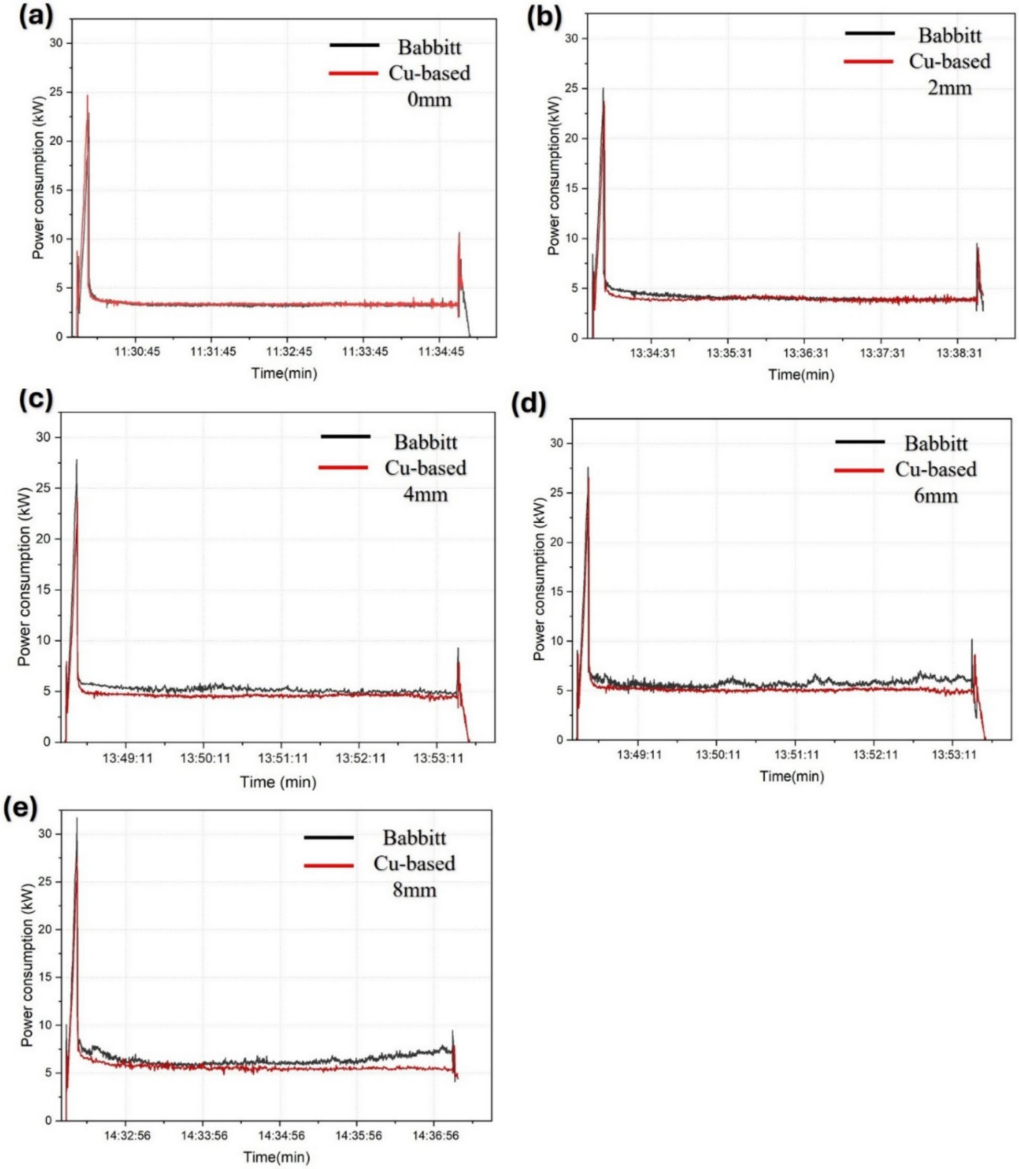
Power consumption of the saw test bench during 5-minute test runs at different arm lengths: (**a**) 2 mm, (**b**) 4 mm, (**c**) 6 mm, and **d** 8 mm for the Babbitt and composite guide pads

Figure 21 illustrates the variation in power consumption of the saw test bench under different operating conditions during a 5-min test. The baseline condition (0 mm arm length) corresponds to the no-load condition, while the subsequent curves represent the system’s power response at increasing arm lengths. As the arm length increases, indicating a higher contact angle, the overall power demand rises. The composite guide pad consistently demonstrates lower power consumption compared to the Babbitt guide pad in all deflection angles.

When the arm length was initially set to 2 mm, only a marginal difference in power demand was observed between the two guide pads, indicating comparable frictional resistance under low contact pressure. However, as the arm length continued to increase, corresponding to a higher contact pressure and more intense interaction between the saw blade and guide pad, the overall power consumption for the Babbitt guide pad rose further. As illustrated in the plots, the Cu-Fe-based composite consistently exhibited lower and more stable power consumption compared with the Babbitt pad throughout the successive test runs. This reduced energy demand can be primarily attributed to the superior lubricating behaviour of the composite guide pad, derived from the presence of solid lubricants. The presence of solid lubricants collectively minimizes interfacial shear stress during sliding. The Babbitt guide pad displays a notably higher and more unstable power consumption profile, particularly at an arm length of 6 mm. This behaviour is likely associated with localized adhesive interactions, cyclic stick–slip phenomena, and the material’s limited capacity to sustain a continuous lubricating film under elevated contact pressures, resulting in transient frictional fluctuations and increased energy dissipation during operation. Increasing the arm length to 8 mm increases the power demand above 5 kW for both materials. However, the Babbitt pad exhibits a secondary rise in power toward the end of the run. This late-stage increase is consistent with thermal softening and melting of the Babbitt, which enlarges the real contact area as illustrated in Fig. 18.

**Fig. 22:**
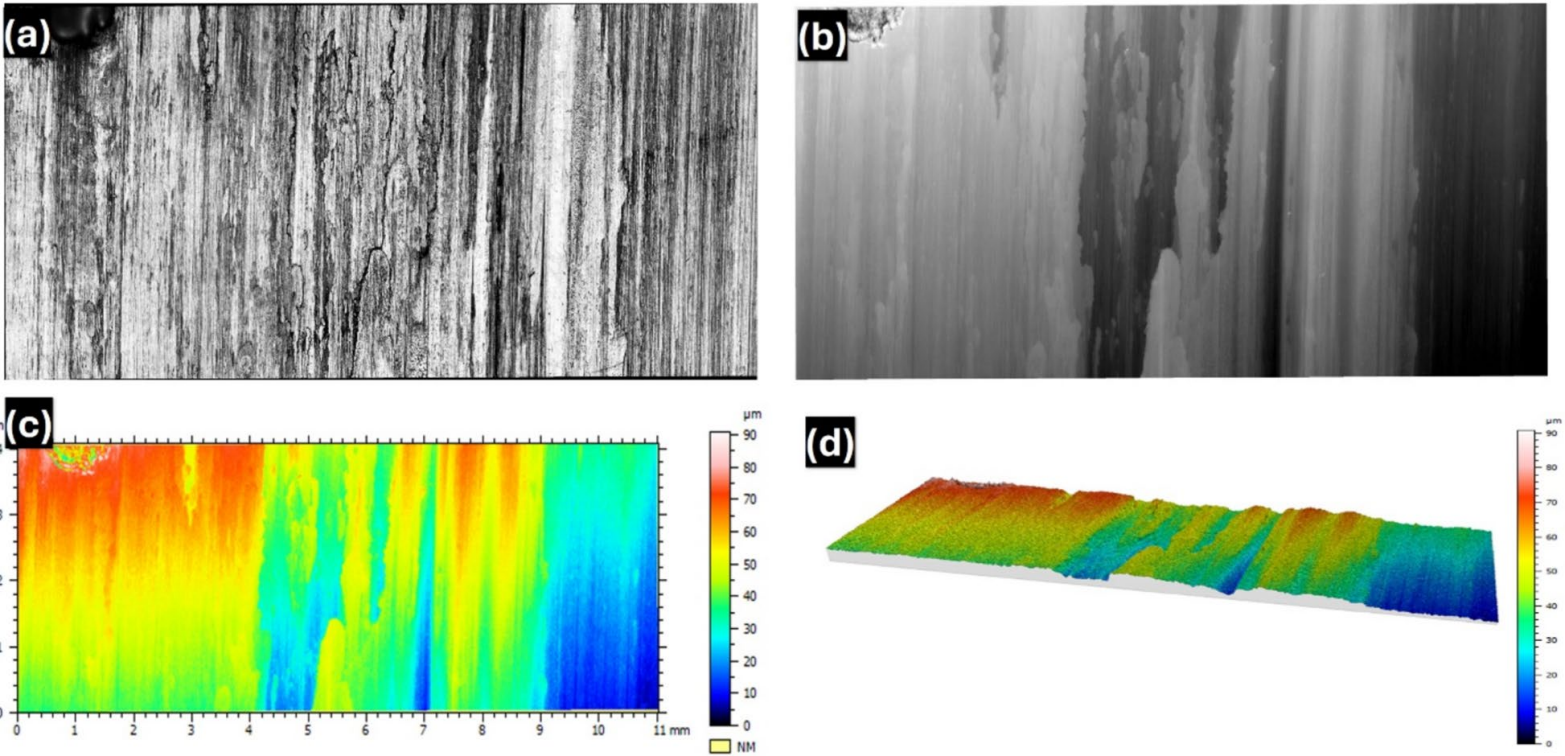
(**a**) Optical micrograph, (**b**) height map, (**c**) pseudo-colour and (**d**) 3D profile of the worn surface of Babbitt

**Fig. 23:**
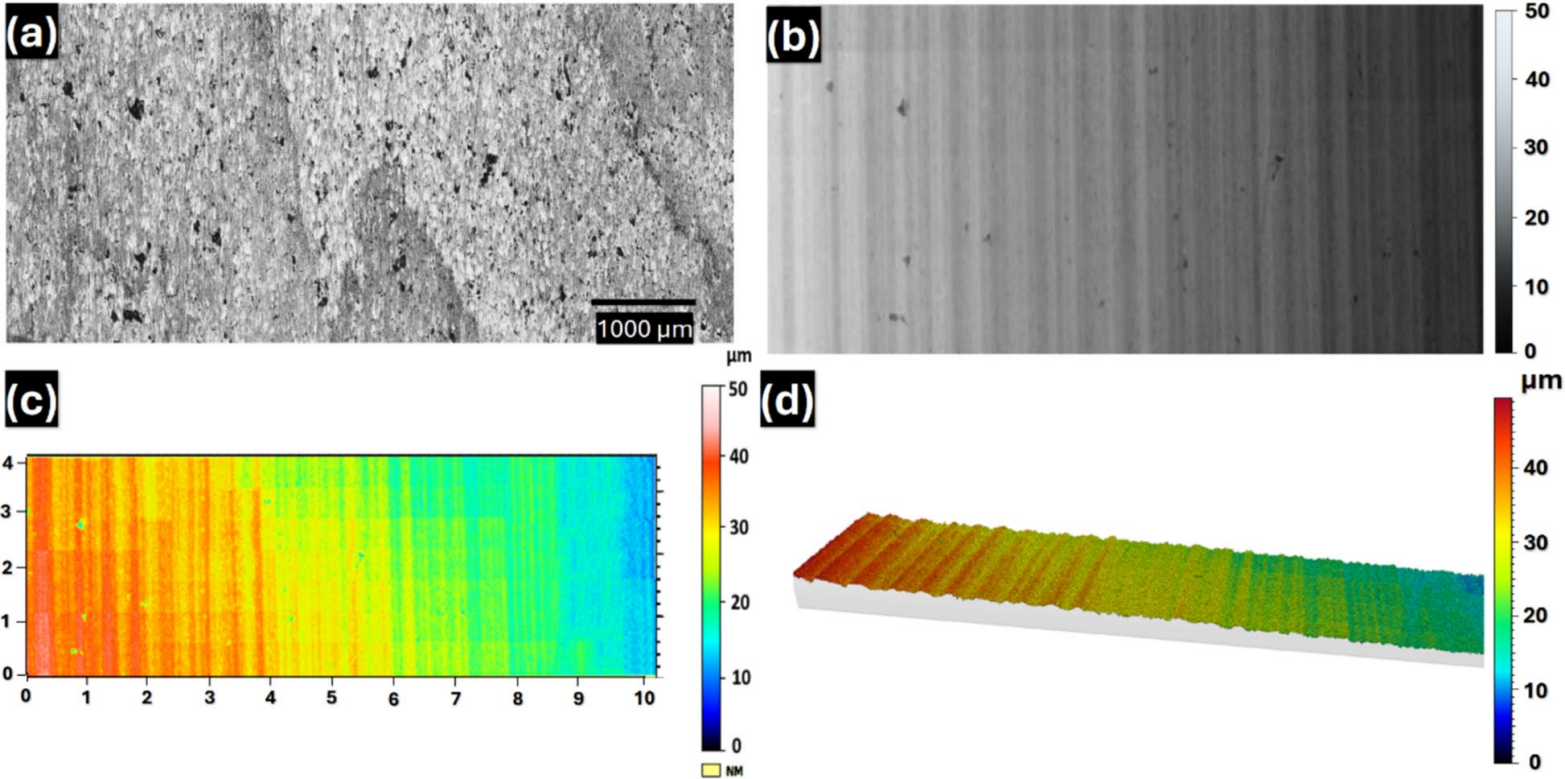
(**a**) Optical micrograph, (**b**) height map, (**c**) pseudo-colour and (**d**) 3D profile of the worn surface oof Cu-Fe composite

Figures 22 and 23 illustrate the distinct differences in surface morphology observed in the regions that experienced the most severe material loss on the Babbitt and composite guide pads following the semi-industrial test. The height map of the worn Babbitt surface presented in Fig. 22b shows severe and non-uniform surface degradation, characterized by deep, continuous longitudinal grooves and significant irregularities in height distribution across the contact region. This highly heterogeneous morphology reflects a progressive deterioration of surface integrity, where sharp peak-to-valley transitions and abrupt topographical variations indicate material ploughing and surface fatigue. Such features are indicative of the alloy’s limited ability to withstand sustained mechanical loading, particularly under elevated contact pressures and high sliding velocities. The loss of surface continuity suggests detachment and re-deposition of wear debris, which further exacerbates surface roughness and compromises the stability of the contact interface. This degradation is further confirmed by the 3D surface reconstruction (Fig. 22d), which reveals a highly uneven and disrupted profile with substantial peak-to-valley amplitudes and extensive zones of material removal and high wear. These topographical features highlight the inability of the Babbitt alloy to maintain structural coherence and a stable tribological regime under prolonged sliding conditions, ultimately leading to progressive surface collapse and accelerated wear propagation.

In contrast, the composite guide pad exhibits a markedly smoother and more homogeneous surface topography, highlighting its superior mechanical robustness and enhanced resistance to wear under identical operating conditions. As illustrated in Fig. 23 both the confocal height map and the corresponding 3D reconstruction reveal lower surface degradation, with shallow, finely grooves and a relatively uniform height profile across the examined region. This interpretation is reinforced by the quantitative surface roughness parameters obtained before and after testing. Table 5 summarizes the surface roughness measured on a representative region of both the Babbitt and composite guide pads before and after testing. Before the semi-industrial tests, the surface roughness of the Babbitt and composite guide pads was nearly identical (Since both guide pads underwent the same surface finishing procedure before testing). However, after the test, the composite guide pad exhibited a significantly lower surface roughness (Sa = 2.01 µm) than the Babbitt pad (Sa = 6.53 µm), indicating a smoother post-test surface. The composite guide pad showed an increase in surface roughness by a factor of approximately 4, whereas the Babbitt material exhibited a much larger increase by a factor of about 14. This lower surface roughness of the composite guide pad further validates the improved wear resistance and superior surface integrity of the composite guide pad. The smoother morphology suggests a stable and well-supported contact interface, where the combined effect of the harder Fe reinforcing phases within the self lubricated matrix contributes to improved load sharing and reduced friction and material loss. The shallow depth and lower density of wear grooves emphasize (Fig. 23) the composite’s capacity to limit material removal and suppress severe deformation. These characteristics demonstrate the ability of the Cu-Fe-based composite to sustain mechanical loads more effectively, thereby ensuring prolonged service life and improved operational stability in demanding industrial conditions.

**Table 5:** Surface roughness of the Babbitt and composite guide pad before and after the test, processed using ConfoMap

	Babbitt surface roughness (μm)	Composite surface roughness (μm)
Before	0.46	0.501
After	6.53	2.014

## Conclusion

This study evaluated the effect of incremental Fe additions on a self-lubricating Cu–5 vol.% NCG–6 vol.% CaF₂ composite as a potential replacement for conventional Babbitt alloy in circular saw guide pad applications. The results demonstrate that Fe incorporation plays a critical role in enhancing both tribological and mechanical performance under laboratory and semi-industrial conditions.

Pin-on-disk and dry sand rubber wheel (DSRW) tests showed that an optimal Fe content of 42 vol.% (P4) significantly reduced wear, achieving approximately 94% reduction in sliding wear and 81% reduction in abrasive wear compared to Babbitt. This improvement is attributed to increased hardness, enhanced load-bearing capacity, and the formation of a stable oxide-rich tribolayer that limits direct metal-to-metal contact. In contrast, excessive Fe content (52 vol.%) led to microstructural inhomogeneity, brittle oxide formation, and increased wear due to delamination, indicating a critical reinforcement threshold. Mechanical testing confirmed a substantial increase in hardness and yield strength, with the P4 composite exhibiting nearly three times higher yield strength than Babbitt.

Semi-industrial testing further validated the superior performance of the optimized composite, demonstrating lower operating temperatures, improved thermal stability, reduced power consumption due to lower friction between saw blade and composite guide pad, and negligible material transfer compared to Babbitt. Surface analyses confirmed severe damage and adhesive wear in Babbitt, while the Cu–Fe composite maintained a smooth and minimally worn surface.

Overall, the Cu–5 vol.% NCG–6 vol.% CaF₂ composite containing 42 vol.% Fe provides an optimal balance of strength, wear resistance, and tribological stability, making it a promising and durable alternative to Babbitt for high-speed circular saw guide pad applications.

## Data Availability

The datasets generated during and/or analyzed during the current study are available from the corresponding author on reasonable request.
